# Propafenone Overdose: From Cardiogenic Shock to Brugada Pattern

**DOI:** 10.5935/abc.20180033

**Published:** 2018-03

**Authors:** Julio Gil, Bruno Marmelo, Luís Abreu, Hugo Antunes, Luís Ferreira dos Santos, José Costa Cabral

**Affiliations:** Serviço de Cardiologia, Centro Hospitalar Tondela, Viseu - Portugal

**Keywords:** Anti-Arrhythmia Agents, Propafenone, Arrhythmias, Cardiac, Calcium Channel Blockers, Toxicity

## Introduction

Propafenone is a class IC antiarrhythmic drug used in the treatment of ventricular and supraventricular arrhythmias.^1-7^ It is primarily a potent sodium channel blocker, yet also exhibits beta-blocking and calcium channel blocking activity.^4,6,7^ Propafenone is able to induce important ECG changes, namely prolongation of the PR interval, bundle branch block, wide QRS and QT intervals, as well as ventricular tachycardia or bradycardia.^3,4^ It may be associated with significant proarrhythmogenic effects, even at therapeutic doses.^2^ A fatal overdose on propafenone is usually attributed to conduction abnormalities, leading to asystole or electromechanical dissociation. The authors describe two clinical cases of propafenone intoxication with life-threatening ECG changes, but with a favorable final outcome.

## Case Report

### Case 1

**Figure 1:**
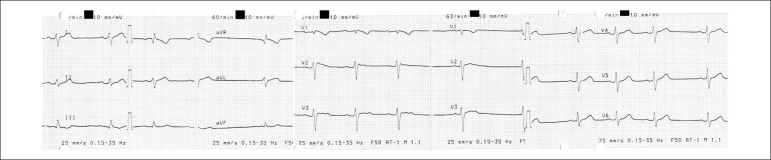
ECG on admission from case 1.

Female patient, 44 years old, with no relevant medical history. The patient was brought to the Emergency Room (ER) following voluntary ingestion of 4500mg of propafenone. While being brought to the ER, the patient had a short-lasting seizure, later regaining consciousness. Upon arrival at the ER, she had a Glasgow Coma Scale (GCS) score of 10 (Eye-3, Motor-5, Verbal-2), bradycardic (55bpm) and hypotensive (Blood Pressure [BP] 85/30mmHg). Clinical examination was unremarkable. Gastric lavage was performed, with removal of what seemed to be pill residuals. Blood work revealed metabolic acidosis. The ECG at admission showed sinus arrhythmia, with right axis deviation, incomplete right bundle branch block (RBBB) and unspecific repolarization changes in DIII, V1 and V2. The PR, QRS and QT intervals were within normal range (Figure 1). BP did not respond to aggressive fluid therapy, thus a dopamine perfusion was started. After approximately an hour of beginning treatment, the patient suffered a tonic-clonic seizure accompanied by extreme bradycardia and widening of the QRS. Unfortunately, due to the urgency of the situation and the critical state of the patient, these electrical changes could not be recorded through standard 12-lead ECG. She was medicated with atropine and benzodiazepine. This resulted in a comatose state (Glasgow scale of 3), worsening of metabolic acidosis and respiratory failure. The patient was intubated, placed on continuous mechanical ventilation and admitted to the Intensive Care Unit (ICU).

Upon admission to the ICU, rhythm strip monitoring revealed atrial fibrillation with occasional sinus activity, along with a widening of the QRS (200 milliseconds) interval. Three hours later, sinus rhythm was restored and QRS interval returned to normal values, with an almost complete disappearance of the RBBB pattern. In the first 6 hours after admission, there was a progressive hemodynamic and clinical stabilization, allowing for a gradual weaning from aminergic and ventilator support. On day two, the patient was conscious and hemodynamically stable. She was discharged after a psychiatric consultation.

### Case 2

**Figure 2:**
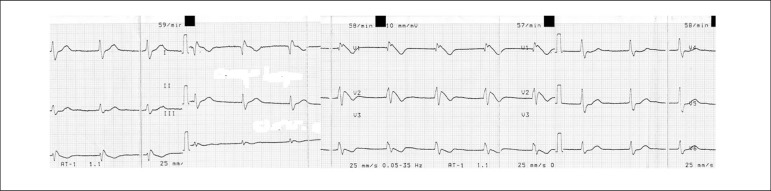
ECG from second clinical case revealing type-1 Brugada pattern.

Female patient, 56 years old, with a history of Atrial Fibrillation and major depressive disorder medicated with propafenone 150 mg twice daily and duloxetine 60 mg once daily. The patient was first observed in a small community hospital after voluntarily ingesting 3000mg of propafenone. At that institution, on arrival, the patient was initially fully awake and gastric lavage was begun. However, shortly afterwards, she developed a tonic-clonic seizure, followed by two episodes of cardiac arrest due to extreme bradycardia. Resuscitation was achieved after less than 2 minutes of advanced life support and atropine administration. After ensuring hemodynamic and electrical stability, the patient was transported to a centralized hospital. Upon admission, she was bradycardic (50 bpm), normotensive (BP 139/89 mmHg), and with a GCS score of 14 (Eye 4, Motor 6, Verbal 4). ECG revealed a junctional rhythm, with a type-1 Brugada pattern in V1 to V3 leads (Figure 2). The patient was admitted to the Cardiac Intensive Care Unit for monitoring. After 24 hours of clinical, hemodynamic and electrical stability, a new ECG was performed, revealing sinus rhythm and disappearance of the Brugada pattern.

## Discussion

Propafenone is a Vaughan Williams Class IC antiarrhythmic agent, and thus a potent sodium channel blocker.^1,3,4,6,7^ It also exhibits beta-blocking and calcium channel blocking activity.^4,6,7^Nearly 100% of propafenone is absorbed. However, because of a first-pass hepatic elimination effect, its bioavailability is unpredictable.^1,4^ Propafenone is metabolized into two major metabolites: 5-hydroxypropafenone and norpropafenone, a process genetically determined by the CYP2D6 enzyme system.^1,4^ The propafenone elimination half-time varies depending on whether the patient is a poor or an extensive metabolizer.^1,4^ There are several infrequent adverse reactions, such as hematologic (agranulocytosis), gastrointestinal, hepatic and neurological (convulsions, amnesia, peripheral neuropathy and exacerbation of myasthenia) reactions.^2^ A number of clinical signs and symptoms have been associated with propafenone intoxication, ranging from nausea and vomiting to seizures, coma, respiratory depression and cardiovascular collapse (Table 1).^4^ Propafenone may be responsible for several ECG changes, including sinus bradycardia, sinus arrest, atrial fibrillation, prolongation of the PR interval, intraventricular conduction abnormalities (QRS and QT widening and right bundle branch block), Brugada pattern,^8-11^ ventricular tachycardia, ventricular flutter or fibrillation and cardiac arrest.^1,4^

**Table 1:** Clinical Signs and Symptoms of Propafenone Intoxication, adapted from Clarot et al^4^

Nausea	Vomiting	Metabolic acidosis		
Blurred vision and sleepiness	Hypotonia	Seizure	Respiratory depression	Coma
Sinus Bradycardia	Sinus arrest	Atrial Fibrillation	AV Block	Intraventricular conduction disorders (QRS widening, Right Bundle Block)
Hypotension	Acute load of the right ventricle	Cardiac failure	Cardiovascular collapse	Cardiac arrest

The authors describe two cases of voluntary ingestion of excessive doses of propafenone, both with successful outcomes. There is no specific treatment. A timely gastric lavage was attempted in both cases. When performed promptly, gastric lavage is the only effective way of eliminating excessive doses of propafenone.^1,3^

In both cases, tonic-clonic seizures were observed. This is an important neurological manifestation of propafenone intoxication.^3,4^ The reason for seizure occurrence is uncertain. Saz et al.^3^ and Clarot et al.^4^ suggest that it may be attributed either to a direct toxic effect of propafenone or to cerebral hypoperfusion caused by arrhythmia or conduction disturbance.

In the first case, all the major clinical warning signs were observed: cardiac failure, conduction disturbance, and seizures.^4^ There was a progressive worsening of the neurological and respiratory status. The patient eventually became comatose, requiring mechanical ventilation. Cardiac failure was also observed, resulting in arterial hypotension, and requiring catecholaminergic support with positive inotropic and vasoconstrictive drugs. After progressive elimination of the drug, weaning from supportive measures was fairly straightforward. Another important aspect is the dynamic ECG changes. The patient underwent rhythm changes (going from sinus arrhythmia to atrial fibrillation and finally returning to normal sinus rhythm) and intraventricular conduction disorders (with widening of the QRS interval and enhancement of the RBBB pattern). These changes occurred only in the first 3 hours after admission, corresponding to peak serum concentration.^4^ This highlights the importance of close monitoring and prompt treatment in the first hours following propafenone overdose.

In the second case, the ingestion of supratherapeutic levels of propafenone revealed a Brugada type 1 pattern on surface ECG. Concealed or intermittent forms of the Brugada Syndrome have been described in a few subset of patients, namely following hyperventilation, beta-adrenergic blockade and alpha-adrenergic stimulation, muscarinic receptors stimulation, and sodium channels blockade inducing or increasing ST elevation.^10,11^ In this particular case, propafenone is able to unmask the concealed Brugada phenomenon due to its sodium channel and beta-adrenergic blocking activity.^10^ The appearance of the Brugada pattern in response to type IC antiarrhythmic drugs does not seem to be associated with a great risk for polymorphic arrhythmias; however, further investigation is needed.^11^ In this case, the Brugada pattern disappeared after drug elimination.

Both patients were closely monitored for 36 to 48 hours. The propafenone elimination half-time ranges from 17 ± 8 hours for poor metabolizers to 5±2 hours for extensive metabolizers,^1,4^ thus monitoring over that period of time is required. Peak serum concentration occurs between 2 and 3 h after ingestion,^4^ during which time the most life-threatening ECG changes may occur.

Both cases are paradigmatic in how unpredictable propafenone overdosing can be. It can range from an almost benign set of symptoms to a catastrophic presentation resulting in death. The first case presented the most important clinical warning signs, namely cardiac failure, conduction disturbance, and seizures. However, thanks to immediate treatment, the patient survived. The second case was critical as well, considering the seizing and extreme bradycardia requiring advanced life support; however, after the initial catastrophic presentation, clinical stability was maintained throughout the following hours. Another interesting aspect was the fact that a type-1 Brugada pattern was revealed. In both cases, no direct treatment for propafenone intoxication was available. Close monitoring and prompt supportive measures are crucial in assuring a good outcome
